# Are Sellar Xanthogranulomas the Climax of a Spectrum of Secondary Inflammatory Reactions to Various Sellar Lesions?

**DOI:** 10.3390/neurosci7030062

**Published:** 2026-05-21

**Authors:** Lennart W. Sannwald, Andrej Pala, Dag Moskopp, Mats L. Moskopp

**Affiliations:** 1 Department of Neurosurgery, Ulm University, Lindenallee 2, 89312 Günzburg, Germany; lennart.sannwald@uni-ulm.de; 2 Department of Neurosurgery, Vivantes Friedrichshain Hospital, 10249 Berlin, Germany; 3 Oncological Rehabilitation, Immanuel Hospital Märkische Schweiz, Brandenburg Medical School Theodor Fontane, 15377 Buckow, Germany; 4 Department of Neurosurgery, Medical Faculty Carl Gustav Carus, Technische Universität Dresden, 01307 Dresden, Germany

**Keywords:** xantogranuloma, sellar surgery, Rathke’s cleft cyst, chronic inflammatory transition

## Abstract

Since 1999, sellar xanthogranulomas (XGs) have been recognized as a distinct pathological entity characterized by cholesterol clefts, macrophages, chronic inflammatory infiltrates with multinucleated giant cells, necrotic debris, and hemosiderin, suggesting a chronic inflammatory process with repetitive intralesional bleeding. This study aims to characterize the clinical phenomenology of the rare XG entity and to explore its correlation with other lesions. A retrospective screening was conducted of 628 sellar surgeries performed during the 2007–2024 period at a major communal hospital in Berlin and 529 surgeries between 2015 and 2024 at Ulm University. Eight XGs were analyzed. Eight XGs (0.6% of 1157 surgeries) showed mixed intra- and suprasellar localization. Visual deficits and endocrinological insufficiencies were the most common symptoms (four out of eight each). Visual recovery was favorable (three out of four complete, one out of four marked improvement), whereas endocrinological recovery was limited (one out of four). One patient experienced recurrence. Intraoperatively, seven out of eight lesions contained characteristic fluid described as ‘golden water of Gdansk.’ Postoperatively, transient arginine vasopressin deficiency occurred in four out of eight patients. The illustrative case demonstrated transformation of a Rathke’s cleft cyst, while two cases were associated with pituitary adenomas. Sellar XGs are benign, chronic inflammatory and hemorrhagic lesions with low recurrence risk. Their frequent association with other sellar pathologies supports a secondary reactive origin.

## 1. Introduction

While pituitary adenomas were first described as distinct tumors of the pituitary gland in 1900 by Carl Benda, the Viennese pathologist Jakob Erdheim distilled a separate pathological entity from a Babylonian confusion of brain cholesteatomas, carcinomas, sarcomas, and granulomas, which he termed hypophyseal duct tumors in 1904 [1]. Later, both Frazier and Cushing coined the embryologically misleading term craniopharyngioma—implying an endodermal origin contrary to the ectodermal origin (of the stomodeum) proposed by Erdheim [2,3].

In 1999, Paulus et al. first described sellar region xanthogranuloma as clinically and pathologically distinct entity in a collective of 110 patients initially diagnosed with craniopharyngioma [4]. Histological hallmarks of xanthogranuloma consist of cholesterol clefts, macrophages, chronic inflammatory infiltrates with multinucleated giant cells, necrotic debris, and hemosiderin deposits suggesting a chronic inflammatory nature with repetitive bleeding into the lesion. This histopathological overlap between craniopharyngioma and xanthogranuloma is underlined by the fact that even Erdheim’s initial description of craniopharyngioma as an entity in 1904 reported secondary inflammatory reactions such as multinucleated giant cells, necrosis, and cholesterol flakes in three out of seven cases [1]. Clinically, xanthogranuloma was associated with early adulthood, primarily intrasellar location, more severe endocrinological impairment, longer preoperative duration of disease, and better resectability, as well as more favorable outcome.

In 1998, Folkerth et al. described xanthomatous hypophysitis as infiltration of normal pituitary by foamy histiocytes and lymphocytes without granulomas, multinucleated giant cells, or hemosiderin in three patients suggesting a close relationship with xanthogranuloma as different stage without granuloma formation and repetitive bleeding in a disease continuum [5]. A common terminological misconception regarding xanthomatous hypophysitis stems from the fact that xanthogranulomatous hypophysitis was defined three years after, in addition and independently from Paulus et al. based on two cases, with very similar criteria of xanthogranuloma to those described by Paulus in 37 cases. Xanthogranulomatous hypophysitis was characterized by foamy macrophages, lymphocytic infiltration, and multinucleated giant cells with granuloma formation without mention of hemosiderin or cholesterol clefts as signs of repetitive bleeding, thus representing an interim or transitional stage between xanthomatous hypophysitis and xanthogranuloma [6].

Furthermore in recent years, xanthogranuloma has also been implied as secondary disease reaction in Rathke’s cleft cysts and pituitary adenomas leading to xanthogranulomatous transformation by chronic inflammation and repetitive bleeding [7,8]. Although xanthogranuloma of the sellar region was introduced as a distinct entity into the WHO classification of central nervous system tumors, xanthogranuloma or cholesterol granuloma has long been known as intracranial disease manifesting at a purely suprasellar location [9], in the middle fossa extradural space [10], the anterior fossa [11], as well as the choroid plexus [12] and the petrous apex [13,14]. Due to its nature as a closed compartment containing the pituitary gland and in direct neighborhood to the visual pathway and hypothalamus, sellar xanthogranulomas display a potential of devastating neurological and endocrinological sequelae even at relatively small size. Nevertheless, whereas intracranial xanthogranulomas outside the sellar region remain rarities and characterization of sellar xanthogranulomas is based on case reports or small case series and is usually omitted from classical textbooks, they may comprise up to 2% of surgically relevant sellar lesions [15]. However, while sellar xanthogranuloma appears to be particularly amenable to surgical resection with low risk of postoperative recurrence [15,16], there is no dependable radiological pattern for pre-histological diagnosis of xanthogranuloma [15,17].

This study aims to contribute eight cases of sellar xanthogranulomas to the literature and report intraoperative hallmarks. Thus, integrating our cases into the body of literature, the secondary inflammatory nature of xanthogranulomas is highlighted, while sellar xanthogranulomas are interpreted as the climax of a continuous spectrum of reactive inflammatory and hemorrhagic changes to primary sellar lesions.

## 2. Materials and Methods

For this two-center retrospective study, 628 sellar surgeries carried out by the senior author (DM) at the neurosurgical department of a major communal hospital in the center of Berlin between 1 April 2007 and 31 January 2023 (contributing 7 cases of XG) as well as 529 sellar surgeries performed at Ulm University District Hospital Günzburg between 1 January 2015 and 31 December 2024 (contributing 1 case of XG) were retrospectively screened for pathological diagnosis of xanthogranuloma. All cases with histopathologically verified sellar xanthogranuloma were included. While the microsurgical transsphenoidal route was carried out by DM, surgeries in Günzburg were performed endoscopically. Afterwards, epidemiological, clinical, radiological, and follow-up data, as well as surgical notes, were acquired by chart review of the resulting eight cases. Scientific analysis and publication of the cases were approved by the institutional ethics committee of Ulm university (identification number 225/24 on 30 April 2019) and informed consent for analysis was waived accordingly due to the retrospective nature of the study.

## 3. Results

### 3.1. Exemplary Case

**Figure 1:**
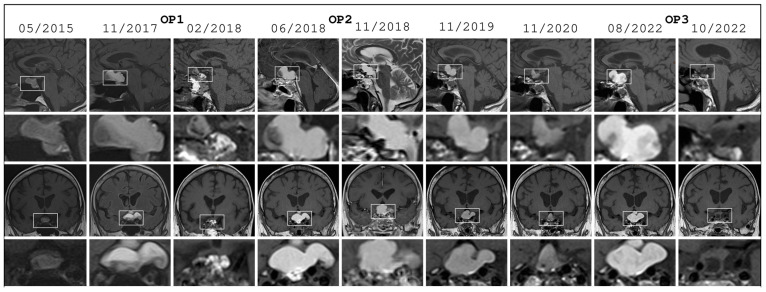
Sequence of MRIs of patient No 1 (exemplary case) over a time course of seven years. The white boxes mark the sella region, which are shown in the corresponding zoomed-in panels. Surgical interventions are marked with OP1–3.

A 68-year-old female patient presented to neurosurgical practice due to an incidental suprasellar predominantly cystic lesion detected on MRI (see a course of images in Figure 1). She suffered from long-standing hypothyroidism, had no visual deficits and no other endocrinological abnormalities. Together with the patient we opted for surveillance and follow-up MRI due to the lack of symptoms and unknown nature of the lesion. The patient was followed through yearly cranial MRIs for seven consecutive years with gradual progression in lesion size without new visual or endocrinological deficits. Ophthalmological examination at the end of that year revealed no field defect and visual acuity of 1.0 in the right and 0.8 in the left eye.

At the beginning of the eighth year of follow-up, the patient suffered from rapidly progressive blurred vision. Visual examination revealed bitemporal hemianopsia and visual acuity of 0.4 in the right and 0.2 in the left eye. Due to the rapid visual deterioration, we opted for optic chiasm decompression through microsurgical cyst fenestration via a transnasal transsphenoidal trans-tuberculum approach. Surgery took 46 min and the intraoperative cerebrospinal fluid leak was primarily treated with an abdominal fat graft and temporary lumbar drain. Intraoperatively, the surgeon reported the typical resemblance of the cystic content to ‘golden water of Gdansk’ (clear fluid containing golden crystals). The patient’s vision improved immediately to an acuity of 0.8 in both eyes and no remaining visual field defect, while postoperative MRI showed complete cyst collapse. Endocrinologically, the patient remained on preexisting thyreotropic substitution without any further endocrinological deficit, especially no postoperative arginine vasopressin deficiency. Pathological evaluation of the partially resected cyst wall revealed a single layer of epithelium consistent with Rathke’s cleft cyst (RCC).

Half a year later, the patient complained about the worsening of her vision again. Ophthalmological evaluation revealed only a slight compromise of visual acuity (0.8 in the right and 0.6 in the left eye), while cranial MRI showed fulminant recurrence of the suprasellar lesion. A second transsphenoidal trans-planum cyst fenestration was carried out in 43 min and intraoperative cerebrospinal fluid leak was primarily covered with fascia lata and treated with temporary lumbar drain. Once again, visual acuity improved instantly to 1.0 in the right and 0.8 in the left eye with desaturation for red color in the left temporal visual field as a sign of mild chiasm syndrome. Additionally, the patient needed continuous cortisol replacement (hydrocortisone 10-0-0 mg/d). There was no postoperative arginine vasopressin deficiency. Pathological examination revealed no signs of typical Rathke’s cleft epithelium but cyst wall infiltration with few macrophages and siderophages. Cranial MRI two months postoperatively showed rapid cyst recurrence without correlating symptoms.

**Figure 2:**
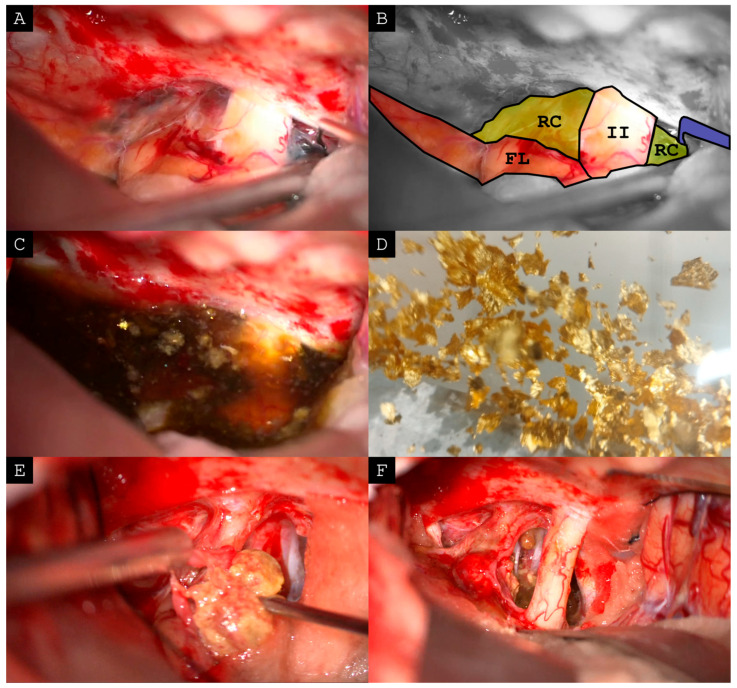
Intraoperative view of the transcranial cyst resection via a frontolateral craniotomy and subfrontal dissection route. (**A**,**B**) Initial view on the pathology. (**C**) After opening the cyst a brownish fluid with characteristic cholesterol clefts drained into the surgical site. (**D**) As a comparison an image of ‘Danziger Goldwasser’ (literal translation ‘golden water of Gdansk’)—a herbal liqueur containing 22-carat gold leaf flakes. (**E**) Also, solid material could be recovered. (**F**): Final operativ field after careful dissection preserving microvascular structures in the presence of scar adhesions of eloquent areas. Operation was performed by DM. (FL = frontal lobe; RC = Rathke cyst; II = optical nerve).

The patient was followed for another four years in which the lesion first slowly decreased in size before erupting again. After 12 years of mostly stable and symptom-free disease, the now 80-year-old patient suffered from her worst visual symptoms yet. Ophthalmological evaluation showed visual acuity of 0.4 in the right and 0.5 in the left eye with bitemporal hemianopsia. Following an extensive discussion with the patient regarding her age, social situation (nursing her debilitated husband), and previous two transnasal operations, we opted for transcranial cyst resection via a frontolateral craniotomy as described by Mario Brock and a subfrontal dissection route [18]. Intraoperatively, the surgeon again reported the drainage of golden water of Gdansk and extensive cyst wall resection (see Figure 2). The patient developed no arginine vasopressin deficiency and showed rapid improvement of vision to 1.0 in both eyes without visual field defect in ophthalmological evaluation. Postoperative cMRI after two weeks demonstrated no signs of a remaining lesion. The third pathological examination was consistent with xanthogranuloma of a Rathke’s cleft cyst. At the latest clinical consultation over two and a half years after the operation the patient showed no new symptoms. She substituted low-dose hydrocortisone and L-thyroxine. The surgical procedure is displayed in Video S1 in Supplementary Materials.

This case is exemplary in several respects: firstly, it demonstrates that the sellar neurosurgeon must think in neurological, ophthalmological, endocrinological, and surgical terms at the same time while committing to long term follow-up of patients. Secondly, the pathological history of this case documents a stepwise change of a Rathke’s cleft cyst into a xanthogranuloma, supporting theories that xanthogranulomas result from a secondary reaction to a different primary disease. Thirdly, this case illustrates the typical intraoperative finding of cystic fluid resembling the ‘golden water of Gdansk’, serving as an intraoperative sign of the nature of the lesion at hand.

### 3.2. Summary of All Eight Cases

Clinical and epidemiological data of all cases is shown in Table 1.

**Figure 3:**
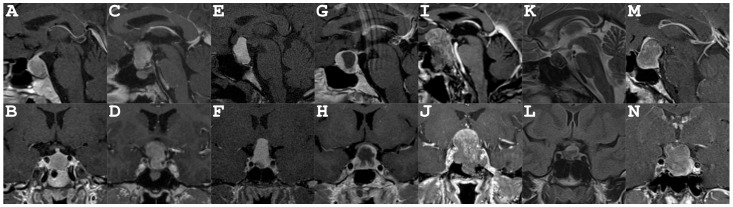
Initial MRI of Patients 2–7 (cf Table 1): (**A**,**B**): Patient 2, (**C**,**D**): Patient 3, (**E**,**F**): Patient 4, (**G**,**H**): Patient 5, (**I**,**J**): Patient 6, (**K**,**L**): Patient 7, (**M**,**N**): Patient 8. Overall lesions showed both intralesions and interlesional heterogeneity.

Three out of eight patients were male and five out of eight were female, while the age ranged from 15 to 70 years. The localization of lesions was purely intrasellar in two out of eight patients, and combined sellar and suprasellar in five out of eight patients with a predominant suprasellar component in four out of five. The exemplary case presented primarily in the suprasellar infradiaphragmatic region with only minor sellar extension (see Figure 3). Four out of eight patients suffered from endocrinological deficits at admission (see Table 1). Four out of eight patients presented with visual deficits preoperatively. In summary, one out of eight patients suffered from both endocrinological and visual deficits, while four out of eight patients suffered from visual deficits, and three out of eight demonstrated endocrinological deficits without visual deficits.

Six out of eight patients underwent microsurgical transsphenoidal resection, one patient received endoscopic transsphenoidal resection with intraoperative MRI, and one patient received microsurgical transsphenoidal cyst fenestration twice followed by transcranial resection. In seven cases out of eight, the lesion discharged typical clear or slightly brownish fluid with golden bodies resembling golden water of Gdansk either after opening of sellar dura or during curettage or splitting of healthy pituitary. Histological examination revealed xanthogranuloma in five out of eight cases, a combination of xanthogranuloma and pituitary adenoma in two out of eight cases, and RCC and xanthogranuloma in one out of eight cases.

Four out of eight patients showed transient arginine vasopressin deficiency postoperatively, all of which recovered. On the other hand, only one out of four patients with preoperative endocrinological deficits demonstrated postoperative recovery (a patient with solitary gonadotropic insufficiency). On the contrary, four out of four patients with visual deficits showed marked improvement, while two out of four patients had no residual visual deficit when leaving the hospital (both with markedly reduced visual acuity without field defect preoperatively). Two patients still suffered from mild temporal visual field defect at discharge both of which completely recovered at the last follow-up. Six out of eight patients showed no recurrence of their sellar lesion after resection, and one out of eight patients retained a remnant of the xanthogranuloma-associated pituitary adenoma in the cavernous sinus (follow-up nine to 180 months as shown in Table 1). While the exemplary case demonstrates a gradual transformation of a RCC into a xanthogranuloma as reflection of a chronic inflammatory and hemorrhagic process.

## 4. Discussion

The pathological hallmarks of sellar xanthogranuloma comprise cholesterol clefts, foamy macrophages, chronic inflammatory infiltrates with multinucleated giant cells, necrotic debris, and hemosiderin deposits suggesting a chronic inflammatory nature with repetitive bleeding into the lesion [4]. However, intraoperatively released fluid in cystic intracranial lesions is not typically seen by pathologists and sellar xanthogranulomas show no predictive characteristics in preoperative MR or CT imaging [19]. This is underlined by the heterogenous imaging characteristics presented in Figure 3. Intralesional fluid resembling “golden water of Gdansk” might guide the surgeons approach to the lesion during surgery indicating a benign lesion with low risk of recurrence and low vascularity. If the surgeon is confident about the diagnosis of xanthogranuloma based on macrosopic aspects, as illustrated in Figure 2, an aggressive resection in the highly eloquent sellar area is not required and may pose unnecessary risk for the patient—while decompression, curettage, and irrigation constitute a valid surgical strategy with favorable outcomes.

Xanthogranulomatous changes are hallmarks of chronic inflammatory lesions in different systems such as xanthogranulomatous pyelonephritis [20], xanthogranulomatous cholecystitis [21], xanthogranulmatous sialadenitis, or benign histiocytoses such as juvenile xanthogranulomas [22]. This implies a reactive nature of the lesion. Although sellar xanthogranulomas were introduced as a distinct [23] entity due to their specific location with associated complex of symptoms and clinical course, the pathological hallmarks suggest a reactive nature of the lesion. In fact, Paulus et al. first and foremost discriminated sellar xanthogranulomas from adamantinomatous craniopharyngiomas, which they were first diagnosed as, thus defining craniopharyngioma diagnosis more precisely [4].

This is supported by accumulating reports of sellar xanthogranulomas associated with other distinct sellar pathologies. In 2013, Amano et al. described seven cases of sellar xanthogranulomas operated on transsphenoidally, five of which were localized intra- and suprasellar while two were pure suprasellar lesions [7]. In six out of seven lesions pathological evaluation revealed components of Rathke’s cleft cysts thus leading the authors to the conclusion that xanthogranulomas present an inflammatory and hemorrhagic degeneration of Rathke’s cleft cysts. Our illustrative case documents the xanthogranulomatous transformation of a Rathke’s cleft cyst that was followed over a course of 13 years with repeated surgical treatment. Interestingly, Amano et al. similarly describe xanthochromic or yellow-brown cyst content in six out of seven cases supporting the sign of “golden water of Gdansk” as an intraoperative hint toward xanthogranuloma. Although Rathke’s cleft cysts show a significant tendency towards recurrence in around 22–26% of cases [24,25], xanthogranulomatous transformation appears to diminish this risk (no recurrence of regrowth in three total, two subtotal, and two partial resections by Amano et al. with 12 to 84 months follow-up). Similarly, Rahmani et al. reported no recurrence among four parasellar xanthogranulomas in a cohort of 643 parasellar lesions with a mean follow-up of 61 months [26]. A recent report by Ehara et al. on RCC associated with secondary hypophysitis might similarly complete the continuum of RCC, RCC with hypophysitis, and RCC with xanthogranulomatous transformation [27].

However, in 2017, Kleinschmidt-DeMasters et al. comprehensively reviewed the association of sellar xanthomatous hypophysitis and sellar xanthogranuloma as a result of a mini-symposium on adamantinomatous and xanthomatous lesions of the sella, adding 26 pathological specimens from 23 patients treated between 2004 and 2017 to the literature [8]. In this study, both xanthomatous hypophysitis and xanthogranuloma showed a significant pathological overlap with ciliated one-layered epithelium and mucinous content suggestive of Rathke’s cleft cysts (xanthomatous hypophysitis with RCC *n* = 8, xanthogranuloma with RCC *n* = 7). Additionally, overlap of xanthogranuloma with xanthomatous hypophysitis (*n* = 3), adamantinomatous cranioharyngioma (*n* = 1), epidermoid (*n* = 1), pituitary adenoma (*n* = 1), and even histological signs of both adamantinomatous craniopharyngioma and RCC (*n* = 1) was reported. In fact, solitary xanthogranuloma was only diagnosed in three out of 26 cases (solitary xanthomatous hypophysitis only once). This reinforces the hypothesis that sellar xanthogranulomas are reactive lesions to sellar pathologies—beyond Rathke’s cleft cysts. Again, this is supported by the fact that in addition to one RCC, two out of the remaining seven xanthogranulomas in our series occurred in pituitary adenomas.

Overall, the histopathological and clinical manifestation presented in these cases, as well as the historical descriptions and definitions, suggest that xanthomatous hypophysitis, xanthogranulomatous hypophysitis, and xanthogranuloma are continuous stages and parts of a spectrum of secondary inflammatory reactions occurring in the sella.

Due to the underlying inflammatory nature of the lesion multiple anti-inflammatory therapies, using corticosteroids for xanthogranuloma is discussed in the literature. A recent report by DeCou illustrates a case where after multiple surgeries the therapy of celecoxib and mycophenolate mofetil prevented the reappearance of previously recurring xanthogranuloma based on hypophysitis [28].

### Limitations

This study of a case series is inherently biased due to the retrospectively collected data and rarity of the lesion, which makes it difficult to estimate a true incidence. All characteristics presented must be interpreted in that context. However, considering the rarity of the lesion this collection of cases represents one of the largest in the literature.

## 5. Conclusions

In summary, accumulating evidence suggests that sellar xanthogranulomas are a secondary inflammatory and hemorrhagic reaction to other sellar pathologies. This study adds eight more xanthogranulomas to the literature, documenting a xanthogranulomatous transformation of a Rathke’s cleft cyst that was operated on three times over 13 years, as well as two xanthogranulomas in pituitary adenomas. ‘Golden water of Gdansk’ is a strong intraoperative hallmark of sellar xanthogranulomas. Sellar xanthogranulomas cause both endocrinological and visual dysfunction and show no reliable radiological pattern. While visual dysfunction responds well to treatment, endocrinological dysfunction improves rarely. Postoperative transient arginine vasopressin deficiency is common, while postoperative recurrence appears to be rare.

## Figures and Tables

**Table 1:** Summary of clinical and epidemiological data of all cases.

No	Age [Years]	Sex	Sellar Extension	Preop Pituitary Deficit	Postop Pituitary Deficit	Preop Visual Deficit	Postop Visual Deficit	CSF Leak	Follow-Up [Month]	Recurrence
1	68	f	Sup	None	ACTH	Bitemp HA, R 0.4, L 0.2	No HA, R 0.8, L 0.8	no	180	Multiple
2	50	f	Intra	None	Temp AVD	None	None	no	132	No
3	28	m	Sup > intra	Panhypo	Panhypo, temp AVD	None	None	no	180	No
4	23	m	Sup > intra	TSH, ACTH, PRL, AVD	TSH, testo	Bitemp HA, R 0.8, L 0.6	No HA, R 1.0, L 0.6	yes	96	No
5	57	f	Sup > intra	None	None	Red desat, R 0.1, L 0.2	No HA, R 1.0, L 1.0	no	84	No
6	70	f	Intra > sup	None	Temp AVD, LH, FSH, PRL	Bitemp HA	Red Desat (only right eye)	no	9	Residuum insight cavernous sinus
7	15	f	Intra	LH, FSH	Temp AVD	None	None	no	24	no
8	46	m	Intra > sup	ACTH, PRL	ACTH, GH LH/FSH	Amblyopia R	No new deficit	no	72	no

(Abbreviations: Bitemp: bitemporal, f: female, HA: hemianopsia, Intra: intrasellar, L: vision of the left eye, m: male, R: vision of the right eye, Red desat: red desaturation, sup: suprasellar, temp AVD: temporary arginine vasopressin deficiency, CSF: cerebrospinal fluid, Panhypo: panhypopituitarism, TSH: thyroid-stimulating hormone, ACTH: adrenocorticotropic hormone, PRL: prolactin, FSH: follicle-stimulating hormone, LH: luteinizing hormone, testo: testosterone, GH: growth hormone).

## Data Availability

Data are available on demand.

## Supplementary Materials

The following supporting information can be downloaded at: https://www.mdpi.com/article/10.3390/neurosci7030062/s1, Video S1: Dissection after frontolateral craniotomy on a subfrontal dissection route. In the final seconds of the video brownish fluid with characteristic cholesterol clefts drain into the surgical site. (The operation was performed by DM. Further preparation is shown in Figure 2).

## Abbreviations

The following abbreviations are used in this manuscript:

RCC	Rathke’s cleft cyst
MRI	magnetic resonance imaging
XG	xanthogranuloma
